# A Fast Sparse Recovery Algorithm for Compressed Sensing Using Approximate *l*_0_ Norm and Modified Newton Method

**DOI:** 10.3390/ma12081227

**Published:** 2019-04-15

**Authors:** Dingfei Jin, Yue Yang, Tao Ge, Daole Wu

**Affiliations:** 1 School of Traffic and Transportation Engineering, Central South University, Changsha 410075, China; dr.kin@csu.edu.cn; 2 The State Key Laboratory of Heavy Duty AC Drive Electric Locomotive Systems Integration, CRRC Zhuzhou Locomotive Co., Ltd., Zhuzhou 412001, China; zdhwdl0722@163.com; 3 China Mobile (Suzhou) Software Technology Co., Ltd., Suzhou 215004, China; getao@cmss.chinamobile.com

**Keywords:** sparse recovery, compressed sensing, approximate *l*_0_ norm, modified Newton method

## Abstract

In this paper, we propose a fast sparse recovery algorithm based on the approximate *l*_0_ norm (FAL0), which is helpful in improving the practicability of the compressed sensing theory. We adopt a simple function that is continuous and differentiable to approximate the *l*_0_ norm. With the aim of minimizing the *l*_0_ norm, we derive a sparse recovery algorithm using the modified Newton method. In addition, we neglect the zero elements in the process of computing, which greatly reduces the amount of computation. In a computer simulation experiment, we test the image denoising and signal recovery performance of the different sparse recovery algorithms. The results show that the convergence rate of this method is faster, and it achieves nearly the same accuracy as other algorithms, improving the signal recovery efficiency under the same conditions.

## 1. Introduction

Compressed sensing (CS) [1,2,3,4] is a theory proposed in recent years. Based on this theory, the original signal can be recovered with sampling values much lower than the Nyquist sampling rate. Assuming that a *m* × 1, signal ***x*** will be recovered from ***y*** = ***Ax***, where$\;x\in R^{n}$,$\;y\in R^{m}$, and $A\in R^{m\times n}$. If *m* < *n*, recovery of ***x*** from ***y*** = ***Ax*** is ill-posed. However, if the ***x*** is *K(K ≤* *m*) sparse, that is, only *K* elements in ***x*** are not 0, then ***x*** can be calculated. ***A***, ***y***, and ***K*** are called measurement matrix, measurement vector, and sparsity level, respectively.

The CS theory consists of two parts. The first is the sparse representation. Because a natural signal is not sparse in the usual case, we need to first make it sparse by mathematical transformation. The second is sparse recovery, which means to recover the sparse signal. Among the two parts, the sparse recovery method directly determines the quality of signal recovery. In this paper, we focus on proposing a sparse recovery algorithm for CS. In the following sections, signal ***x*** is set to be a sparse signal vector.

Generally, all sparse recovery methods are devoted to solve Equation (1) [1,3,5], where ${||x||}_{0}$ denotes the number of nonzero elements in ***x***. Equation (1) is aimed at seeking the minimal ${||x||}_{0}$ solution of the underdetermined linear system of equations ***y*** = ***Ax*** with known ***A*** and ***y***.

(1) $$\{\begin{array}{l} \min{\left|\left|x\right|\right|}_{0}\\ s.t.\;y=Ax \end{array}$$

Clearly, the direct *l*_0_ norm minimization problem is NP-hard [6,7,8], so there are many proposed algorithms to approximate its solution. In general, these methods can be classified into three categories: (1) convex relaxation method (CR) [9,10], (2) greedy algorithm (GA) [11,12], and (3) approximate *l*_0_ norm algorithm (AL0) [13,14].

The CR methods, also called *l*_p_ norm minimization, use the *l_p_* norm to approximate the *l*_0_ norm, which can be shown as Equation (2):

(2) $$\{\begin{array}{l} \min{\left|\left|x\right|\right|}_{p}^{p}\\ s.t.\;y=Ax \end{array}$$

where ${\left|\left|x\right|\right|}_{p}={\left(\sum_{i\;=\;1}^{N}{\left|x_{i}\right|}^{p}\right)}^{1/p}$, and *x_i_* is the element in ***x***. In particular, when *p* = 1, this method is called basis pursuit (BP) algorithm. The performance of this algorithm is stable. However, because it is solved by linear programming without solving the linear equations directly, its computational cost is very high [15]. 

The GA algorithms, such as matching pursuit [12] (MP) and orthogonal matching pursuit [11] (OMP), do not always guarantee a global optimal solution [5]. 

The AL0 adopts a smoothed function with parameters to approximate the *l*_0_ norm of ***x***. Thus, the *l*_0_ norm minimization problem can be converted into the smoothed function minimization problem, which is called the recovery algorithm using the approximate *l*_0_ norm. For instance, Mohimani [14] adopted the following equation to approximate the *l*_0_ norm:

(3) $$f\left(x_{i}\right)=\exp\left(-\frac{x_{i}^{2}}{2\delta^{2}}\right)$$

where *δ* is a positive number approaching 0. Then, the minimization problem can be converted to the following equation:

(4) $$\{\begin{array}{c} \min\overset{N}{\underset{i=1}{\sum }}\exp\left(-\frac{x_{i}^{2}}{2\delta^{2}}\right)\\ s.t.\;y=Ax \end{array}$$

Algorithms of this category directly solve the linear equations in the process of iteration and do not need to use linear programming. Therefore, this algorithm has fast convergence speed and guarantees global optimum. This algorithm solves the defects of CR and GA to a certain degree and has gradually become a popular sparse recovery algorithm, but it cannot avoid the jaggies in the iterative process. That is to say, the convergence direction does not always follow the descending direction of the gradient.

Based in the idea of AL0, we adopt a simple fractional function to approximate the *l*_0_ norm, and, to avoid jaggies in the iterative process, we propose a modified Newton method, which can make the algorithm converge faster. In addition, we neglect the zero elements in the process of computing, which further reduces the amount of computation. Finally, we compare the performance of the proposed algorithm with several typical sparse recovery algorithms in the field of signal recovery and image processing to prove the advantages of FAL0.

## 2. Fast Sparse Recovery Algorithm Using Approximate *l*_0_ Norm

In this section, we first adopt a simple function to approximate the *l*_0_ norm and then derive the process of solving this problem in detail. In addition, we summarize the procedure of the algorithm and analyze its computational complexity.

### 2.1. Implementation of the Algorithm

We adopt Equation (5) to substitute ${||x||}_{0}$

(5) $$f_{\delta }\left(x_{i}\right)=\frac{\delta }{\delta +x_{i}}$$

where *δ* is a positive parameter and, obviously,

(6) $$\underset{\delta \to 0}{\lim}f_{\delta }\left(x_{i}\right)=\{\begin{array}{c} 1,\;x_{i}=0\\ 0,\;x_{i}\neq 0 \end{array}$$

Then, we consider

(7) $$\{\begin{array}{c} \max\overset{N}{\underset{i=1}{\sum }}f_{\delta }\left(x_{i}\right)\\ s.t.\;y=Ax \end{array}$$

When *δ* approaches 0, the optimal solutions of Equation (7) and Equation (1) are same. This problem is an optimization problem with an equality constraint. The Lagrange multiplier method can transform such problems into unconstrained optimization problems, thus avoiding solving linear programming and greatly reducing the computation cost. The detailed process of this method is as follows:

(8) $$\max L\left(x\right)={\left|\left|Ax-y\right|\right|}_{2}^{2}+\lambda \overset{N}{\underset{i\;=\;1}{\sum }}f_{\delta }\left(x_{i}\right)$$

where *λ* is the regularization parameter, which is used to adjust the weight of the two terms in Equation (8) and can be chosen by the method of generalized cross-validation (GCV) [16]. Here, we adopt the Newton method to solve Equation (8), and the convergence direction (Newton direction) ***d*** is shown by Equation (9).

(9) $$d=-{\left[\nabla^{2}L\left(x\right)\right]}^{-1}\nabla L\left(x\right)$$

where ${\left[\nabla^{2}L\left(x\right)\right]}^{-1}$ is the inverse matrix of $\left[\nabla^{2}L\left(x\right)\right]$. In Equation (9)

(10) $$\nabla L\left(x\right)=\frac{\partial L}{\partial x}=2A^{T}\left(Ax-y\right)+\lambda {\left(-\frac{\partial f}{\partial x_{1}},-\frac{\partial f}{\partial x_{2}},\ldots ,-\frac{\partial f}{\partial x_{N}}\right)}^{T}$$

(11) $${\left(-\frac{\partial f}{\partial x_{1}},-\frac{\partial f}{\partial x_{2}},\ldots ,-\frac{\partial f}{\partial x_{N}}\right)}^{T}={\left(-\frac{\delta }{{\left(\delta +x_{1}\right)}^{2}},-\frac{\delta }{{\left(\delta +x_{2}\right)}^{2}},\ldots ,-\frac{\delta }{{\left(\delta +x_{N}\right)}^{2}}\right)}^{T}$$

(12) $$\begin{array}{ll} \nabla^{2}L\left(x\right)=\frac{\partial \left(\frac{\partial L}{\partial x}\right)}{\partial x} & =\frac{\partial^{2}L}{\partial x^{2}}=2A^{T}A+\lambda \mathrm{diag}\left(\frac{\partial^{2}f}{\partial x_{i}^{2}}\right)\\ & =2A^{T}A+\lambda \mathrm{diag}\left(\frac{2\delta }{{\left(\delta +x_{i}\right)}^{3}}\right) \end{array}$$

where ***A****^T^* is the transpose of ***A***.

Clearly, the Hessian matrix $\nabla^{2}L\left(x\right)$ is not always positive definite, which often leads to “jaggies” in the iterative process. This means that the Newton direction is not always gradient descending. A common means to deal with such a problem is to find an approximate matrix that is positive definite to replace the Hessian matrix. The basic idea of this method is to ensure that the objective function converges to the optimal direction regardless of the speed of convergence. This method is called modified Newton method, and the details of it are shown as follows. 

We construct a new matrix

(13) $$E=\nabla^{2}L\left(x\right)+\lambda \varepsilon I$$

where ***ε*** is a set of appropriate elements, and ***I*** is an identity matrix, which can form the elements on the principal diagonal of ***E***.

(14) $$\varepsilon_{i}\;=\;\frac{2x_{i}}{{\left(\delta +x_{i}\right)}^{3}}$$

where *ε_i_* are the elements in ***ε***. Then, we get a positive definite matrix

(15) $$E=2A^{T}A+\lambda \mathrm{diag}\left(\frac{2}{{\left(\delta +x_{i}\right)}^{2}}\right)$$

thus ***d*** is updated to

(16) $$d=-E^{-1}\nabla L\left(x\right)$$

and the recurrence formula of FAL0 is

(17) $$x_{k+1}=hd+x_{k}$$

where *h* is the step length of the iteration, which can be chosen by the line search method [17]. 

Because ***x*** is a sparse vector and most of its elements are 0, we neglect the zero elements to simplify the calculation. Here, we consider

(18) $$S=\left\{i:|x_{i}>0|\right\}$$

(19) $$x^{S}=\left(x_{i}\right),\;i\in S$$

(20) $$A^{S}=\left(a_{i}\right),\;i\in S$$

where ***a****_i_* is the *i*th column of ***A***. Thus, Equation (17) is updated to

(21) $$\{\begin{array}{c} x_{k+1}^{s}=x_{k}^{s}+hd\\ x^{\bar{s}}=0 \end{array}$$

where $\bar{s}$ is the complement set of *S*. In the iterative process, *δ* decreases gradually, and its update formula is *δ_k_*_+1_ = 0.7*δ_k_*.

### 2.2. Algorithm Description

The pseudo code of FAL0 is shown in Table 1, where ***x***_0_ and *δ*_0_ are the initial value of ***x*** and *δ*, respectively. ***A****^T^*(***AA****^T^*)***y*** is the least square solution of ***y*** = ***Ax***. ***A***, ***y***, and *K* depend on the specific sparse problems, and *λ* and *h* can be chosen by GCV and line search method, respectively. The usage of *δ_k_*_+1_ = 0.7*δ_k_* makes *δ* smaller and smaller, which makes the search scope clearer.

### 2.3. Computational Complexity Analysis of FAL0

The FAL0 algorithm is mainly based on the modified Newton method, which is quadratic convergent [18]. The main computational complexity of FAL0 lies in the computation of the initial solution and the iteration, which involves the product of the matrix and vector. The computational burden of ***A***^T^***y*** does not exceed *O*(*n*^2^), and because we neglected 0 in ***x*** in the calculation process, the computational burden will be further reduced.

## 3. Computer Simulation Experiments and Analysis

In this section, we give several computer simulation experiments on the recovery of both random sparse signals and noisy image tasks to prove the performance of FAL0. The performances are compared with BP [9], OMP [11], MP [12], AL0 [14], and AL0-L [13], and these comparison algorithms all refer to the source code given by the author in the supplement. All of the experiments are implemented in MATLAB 2012a on a personal computer with a 2.59 GHz Intel Pentium dual-core processor and 2 GB memory running the Microsoft Windows XP operating system (Redmond, WA, USA).

### 3.1. Simulation Experiments on Random Sparse Signal Recovery

In these simulation experiments, the matrix ***A*** is a Gaussian random matrix of size *m* × *n*, where *m* < *n*. The vector ***x*** is a sparse signal of *n* dimensions. The *K* nonzero elements are randomly distributed in ***x***, and their values obey the standardized normal distribution. The vector ***y*** is given by ***y*** = ***Ax***. When ***y*** and ***A*** are known, we use the above six algorithms to recover ***x***, and the result is x*. 

Parameters *λ* and *h* can be chosen by GCV [16] and line search [17] method, respectively. When the equation set changes, *λ* and *h* will also change.

The sparse recovery algorithm is usually evaluated by precision and convergence speed, and we adopt exact recovery rate and maximal time consumption to indicate these two metrics. 

We assume that if x* satisfies ||***x*** − x*||_2_ ≤ 10^−4^||***x***||_2_, ***x*** is exactly recovered. Thus, the exact recovery rate can be defined as the ratio of the number of the exactly recovered trails to the total number of the trails under the condition of the fixed parameters. All curves in the figures are obtained by averaging the results of 500 independent trails. In addition, the maximal time consumption is defined as the maximal runtime of the 500 trails under the condition of the fixed parameters.

**Figure 1:**
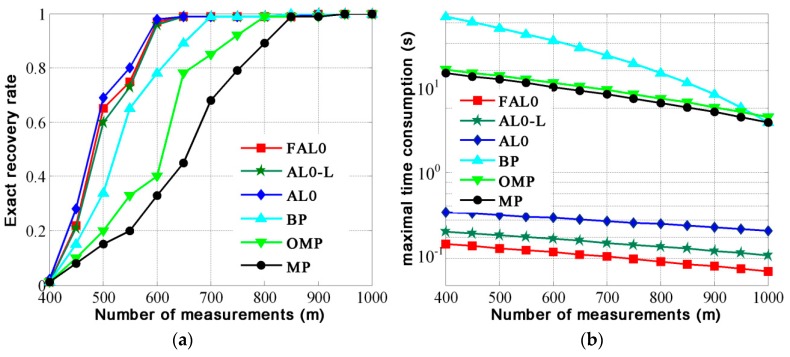
Performance of the six algorithms with different measurements. (**a**) Exact recovery rate; (**b**) maximal time consumption.

In the first experiment, we set *K* = 300 and *n* = 1500, and *m* varies from 400 to 1000. Every time *m* changes, we calculate the exact recovery rate of the six algorithms by averaging the results of the 500 trails. In addition, we show maximal time consumption of six algorithms. The results are shown in Figure 1. The number of measurements means the number of rows of matrix ***A*** and also means the number of elements in vector ***y***. 

**Figure 2:**
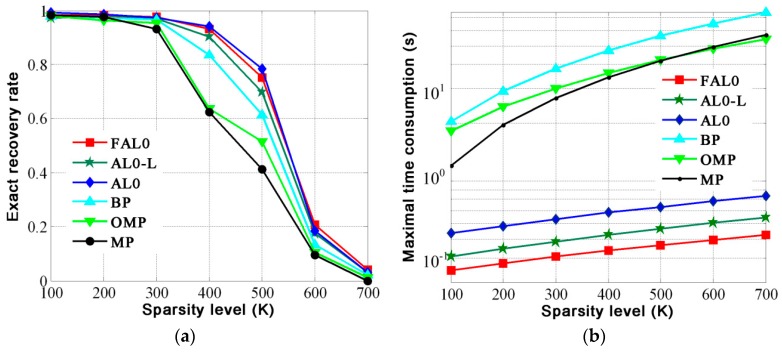
Performance of the six algorithms with different sparsity levels. (**a**) Exact recovery rate; (**b**) maximal runtime time consumption.

In the second experiment, we set *m* = 700 and *n* = 1500, and *K* varies from 100 to 700. Every time *K* changes, we calculate the exact recovery rate of the six algorithms by averaging the results of the 500 trails. In addition, we show maximal time consumption of six algorithms. The results are shown in Figure 2. 

From Figure 1a, we can see that the exact recovery rate of FAL0 is better than that of BP, MP, and OMP and similar to that of AL0 and AL0-L in the case of the same measurement value. From Figure 1b, we find that the maximal time consumption of FAL0 is much less than that of the other five algorithms under the same conditions. 

From Figure 2a, we can see that the exact recovery rate of FAL0 is better than that of BP, MP, and OMP and quite close to that of AL0 and AL0-L in the case of the same sparsity level. From Figure 2b, we can see that maximal time consumption of FAL0 is much less than that of the other five algorithms under the same sparsity level. 

These experiments prove that FAL0 performs well in the aspect of algorithm’s precision and convergence speed, which indicates that the proposed algorithm is suitable for the fast recovery of sparse signals.

### 3.2. Simulation Experiments on Image Denoising

Image denoising is also a common application of sparse recovery algorithm [19]. The detailed process of the CS-based image denoising method can be found in the literature [20], which includes the image’s sparse representation, recovery of the sparse image, and inverse transformation of the sparse image.

In this section, we compare the performance of these six algorithms in the CS-based image denoising method and adopt signal noise radio (SNR, Equation (22)), time consumption, and memory usage to evaluate the performance of these six algorithms. In the experimental process, we keep the other conditions fixed and only change the sparse recovery algorithm.

(22) $$NR=10\cdot \mathrm{lg}\frac{\sum_{i}|x_{i}{|}^{2}}{\sum_{i}|x_{i}^{*}-x_{i}{|}^{2}}$$

In Equation (22), *x_i_* and $x_{i}^{*}$ are the elements in ***x*** and x*, respectively. The higher the SNR, the better the image quality is.

**Figure 3:**
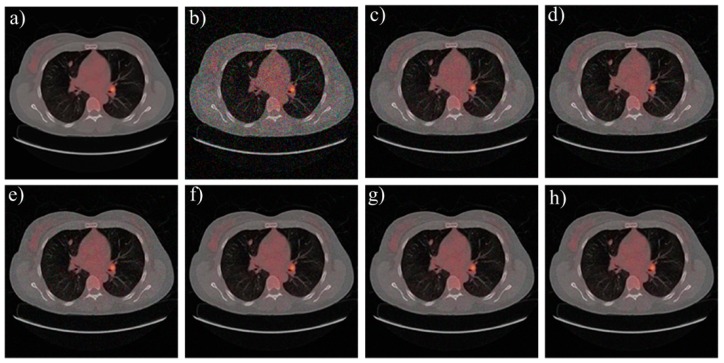
Denoising performance of the six algorithms to the CT image. (**a**) Original image; (**b**) noisy image; (**c**) MP; (**d**) OMP; (**e**) BP; (**f**) FAL0; (**g**) AL0; (**h**) AL0-L.

**Figure 4:**
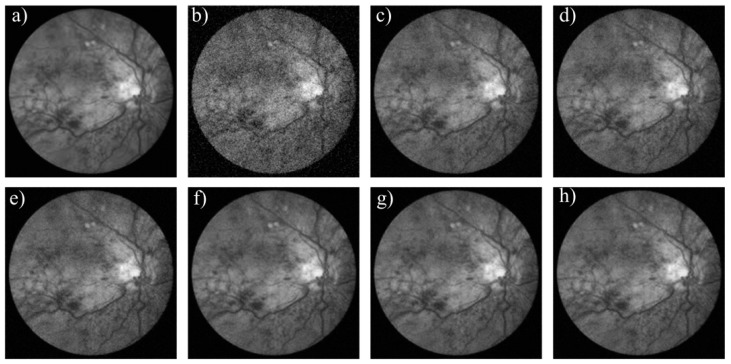
Denoising performance of the six algorithms to the fundus image. (**a**) Original image; (**b**) noisy image; (**c**) MP; (**d**) OMP; (**e**) BP; (**f**) FAL0; (**g**) AL0; (**h**) AL0-L.

**Figure 5:**
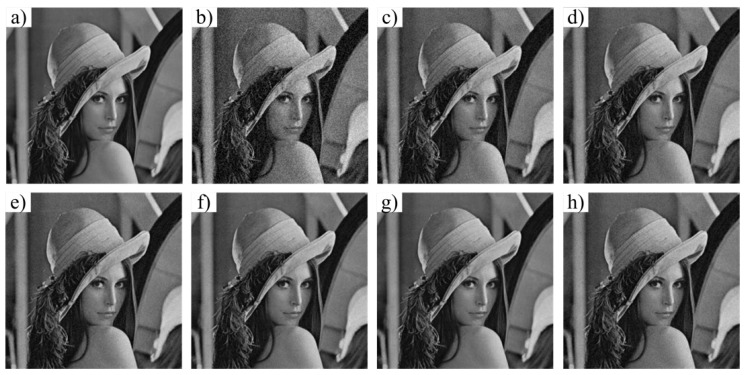
Denoising performance of the six algorithms to the Lena image. (**a**) Original image; (**b**) noisy image; (**c**) MP; (**d**) OMP; (**e**) BP; (**f**) FAL0; (**g**) AL0; (**h**) AL0-L.

We adopt biomedical imaging, including a computer tomography (CT) image and a fundus image, and the typical Lena image as the test objects. Figure 3, Figure 4 and Figure 5 show the denoising effect of these six algorithms intuitively. We can see that the denoising performance of FAL0 is close to that of AL0 and AL0-L but better than that of MP, OMP, and BP.

Their SNR and time consumption are shown in Table 2. From Table 2, we can see that the performance in the application of image denoising of FAL0 is better than that of BP, MP, and OMP and quite close to that of AL0 and AL0-L. Moreover, FAL0 takes the least time, which proves that FAL0 is a fast and effective sparse recovery algorithm.

## 4. Conclusions

Based on the previous study of the approximate *l*_0_ norm, we present a fast and effective sparse recovery algorithm for compressed sensing. We adopt a simple fractional function to approximate the *l*_0_ norm and use the modified Newton method to implement the algorithm, which combines the advantages of fast convergence of AL0 and high accuracy of the Newton method. The results of computer simulation experiments indicate that FAL0 is fast and effective in the application of signal recovery and image denoising.

## Figures and Tables

**Table 1:** The pseudo code of FAL0.

**Algorithm**: FAL0
**Input**: ***A***, ***y***, *λ*, *h*, *K*, and the termination condition: *δ*_min_ = 10^−3^*δ*_0_; **Output**: The recovery signal x*; **Initialization**: ***x***_0_ = ***A**^T^*(***AA**^T^*)***y***, *δ*_0_ = 1; **Step 1**: $S=\left\{i:|x_{i}>0|\right\}$ $x^{S}=\left(x_{i}\right),i\in S$ $A^{S}=\left(a_{i}\right),i\in S$ **Step 2**: Update ***d*** according to Equation (16); **Step 3**: Iterate, $$\{\begin{array}{c} x_{k+1}^{s}=x_{k}^{s}+hd\\ x^{\bar{s}}=0 \end{array};$$ **Step 4**: Update *δ*, *δ_k_*_+1_ = 0.7*δ_k_*; **Step 5**: If *δ* < *δ*_min_, output to x*, or else go to **Step 1** and continue iterating.

**Table 2:** Denoising performance of the six algorithms.

Image	Algorithm	SNR (dB)	Time (s)	Memory Usage (MB)
CT	MP	26.30	46.58	65.76
	OMP	26.19	43.72	60.58
	BP	28.32	78.33	101.75
	FAL0	33.19	10.79	22.37
	AL0	34.01	22.24	36.68
	AL0-L	32.17	15.76	33.52
Fundus	MP	27.52	44.57	68.32
	OMP	26.88	42.16	61.07
	BP	29.11	81.15	112.59
	FAL0	33.12	11.70	20.45
	AL0	33.45	21.12	37.95
	AL0-L	32.77	15.69	36.58
Lena	MP	28.22	49.02	68.70
	OMP	27.73	46.33	59.42
	BP	29.45	85.19	127.34
	FAL0	33.20	11.15	26.82
	AL0	31.54	23.56	38.96
	AL0-L	32.54	18.77	35.40
